# Crystal structure of 4,4′-dimeth­oxy-2,2′-bi­pyridine

**DOI:** 10.1107/S2056989015013985

**Published:** 2015-07-31

**Authors:** Yukiko Kusano, Keiji Ohno, Takashi Fujihara

**Affiliations:** a Department of Chemistry, Graduate School of Science and Engineering, Saitama University, Shimo-Okubo 255, Sakura-ku, Saitama 338-8570, Japan; b Comprehensive Analysis Center for Science, Saitama University, Shimo-Okubo 255, Sakura-ku, Saitama 338-8570, Japan

**Keywords:** crystal structure

## Abstract

In the title compound, C_12_H_12_N_2_O_2_, the dihedral angle between the planes of the two pyridine rings is 5.8 (1)°. Neighbouring mol­ecules are linked *via* C(Me)—H⋯N inter­actions, generating a two-dimensional sheet structure; C—H⋯π inter­actions further link the mol­ecules into a three-dimensional network. An overlapped arrangement of parallel pyridine rings in neighbouring mol­ecules [centroid-to-centroid distance = 3.6655 (15) Å] is observed in the crystal structure.

## Related literature   

For related structure of 4,4′-substituted 2,2′-bi­pyridines, see: Merritt & Schroeder (1956 ▸); Tynan *et al.* (2003 ▸); Pearson *et al.* (2004 ▸); Haberecht *et al.* (2005 ▸); Fujihara *et al.* (2005 ▸). For hydrogen-bonded motifs, see: Etter *et al.* (1990 ▸); Bernstein *et al.* (1995 ▸).
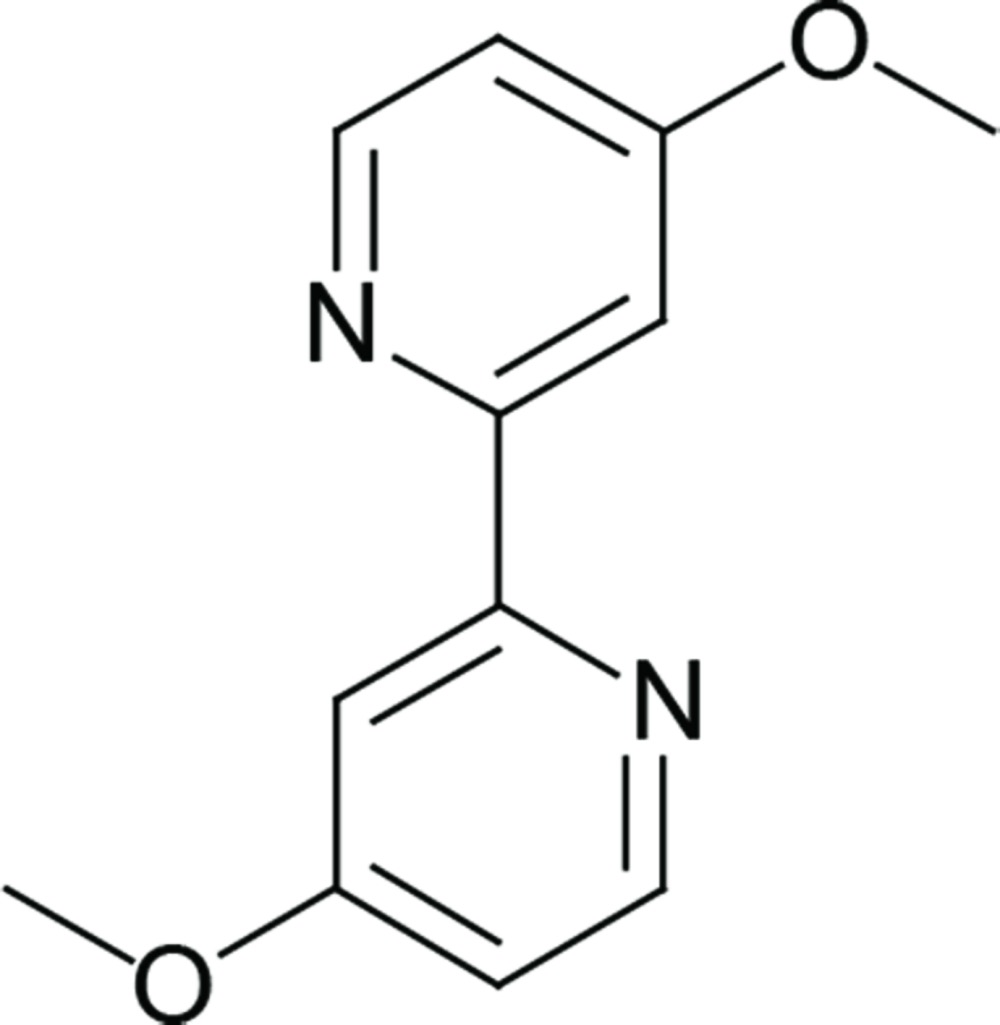


## Experimental   

### Crystal data  

C_12_H_12_N_2_O_2_
*M* *_r_* = 216.24
Monoclinic, 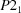

*a* = 6.4235 (11) Å
*b* = 10.8139 (18) Å
*c* = 8.0123 (14) Å
β = 109.462 (2)°
*V* = 524.76 (16) Å^3^
*Z* = 2
Mo *K*α radiation
μ = 0.10 mm^−1^
*T* = 200 K
0.24 × 0.07 × 0.05 mm

### Data collection  

Bruker APEXII CCD area-detector diffractometer
Absorption correction: multi-scan (*SADABS2014*; Bruker, 2014 ▸) *T*_min_ = ?, *T*_max_ = ?
5925 measured reflections
2301 independent reflections
2155 reflections with *I* > 2σ(*I*)
*R*_int_ = 0.014

### Refinement  

*R*[*F* ^2^ > 2σ(*F* ^2^)] = 0.033
*wR*(*F* ^2^) = 0.084
*S* = 1.01
2301 reflections
147 parameters
1 restraint
H-atom parameters constrained
Δρ_max_ = 0.20 e Å^−3^
Δρ_min_ = −0.16 e Å^−3^
Absolute structure: Flack *x* determined using 976 quotients [(*I* ^+^) − (*I* ^−^)]/[(*I* ^+^) + (*I* ^−^)] (Parsons *et al.*, 2013 ▸)
Absolute structure parameter: 0.7 (3)

### 

Data collection: *APEX2* (Bruker, 2014 ▸); cell refinement: *SAINT* (Bruker, 2014 ▸); data reduction: *SAINT* and *XPREP* (Bruker, 2014 ▸); program(s) used to solve structure: *SHELXS97* (Sheldrick, 2008 ▸); program(s) used to refine structure: *SHELXL2014* (Sheldrick, 2015 ▸); molecular graphics: *ORTEP-3 for Windows* (Farrugia, 2012 ▸); software used to prepare material for publication: *XCIF* (Bruker, 2014 ▸).

## Supplementary Material

Crystal structure: contains datablock(s) global, I. DOI: 10.1107/S2056989015013985/xu5860sup1.cif

Structure factors: contains datablock(s) I. DOI: 10.1107/S2056989015013985/xu5860Isup2.hkl

Supporting information file. DOI: 10.1107/S2056989015013985/xu5860Isup3.cml

**Fig. 1.**
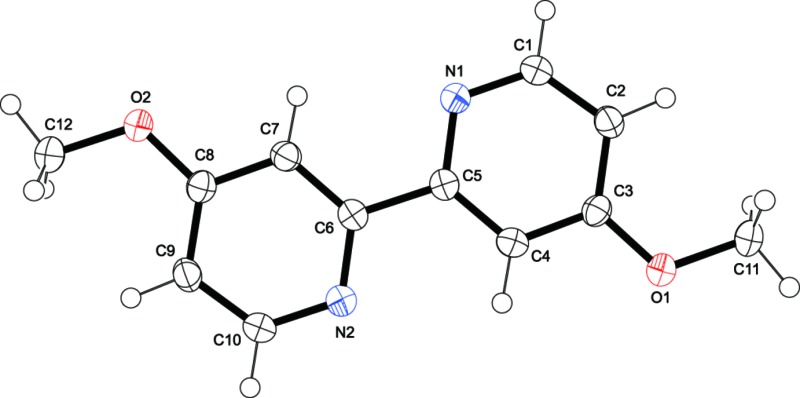
The molecular structure of the title compound, showing the atom labelling. Displacement ellipsoids are drawn at the 50% probability level.

**Fig. 2.**
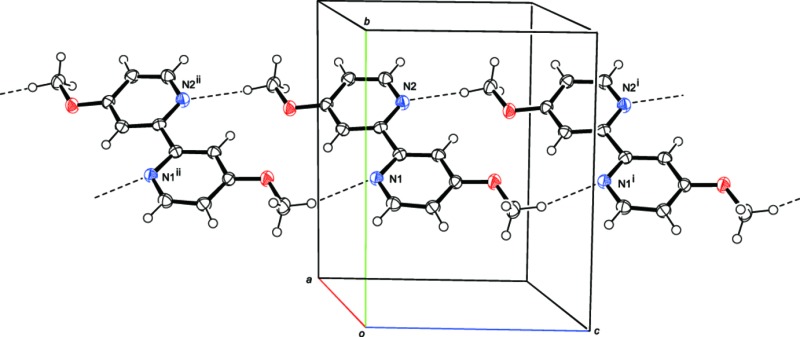
Part of the crystal structure of compound (I) showing the formation of 2D sheet. Dashed lines indicate the intermolecular C-H···N interactions. [Symmetry code: (i) x, y, 1+ z; (ii) x, y, -1+ z.]

. DOI: 10.1107/S2056989015013985/xu5860fig1.tif
The mol­ecular structure of the title compound, showing the atom labelling. Displacement ellipsoids are drawn at the 50% probability level.

x y z x y z . DOI: 10.1107/S2056989015013985/xu5860fig2.tif
Part of the crystal structure of compound (I) showing the formation of 2D sheet. Dashed lines indicate the inter­molecular C-H⋯N inter­actions. [Symmetry code: (i) *x*, *y*, 1 + *z*; (ii) *x*, *y*, −1 + *z*.]

**Fig. 3.**
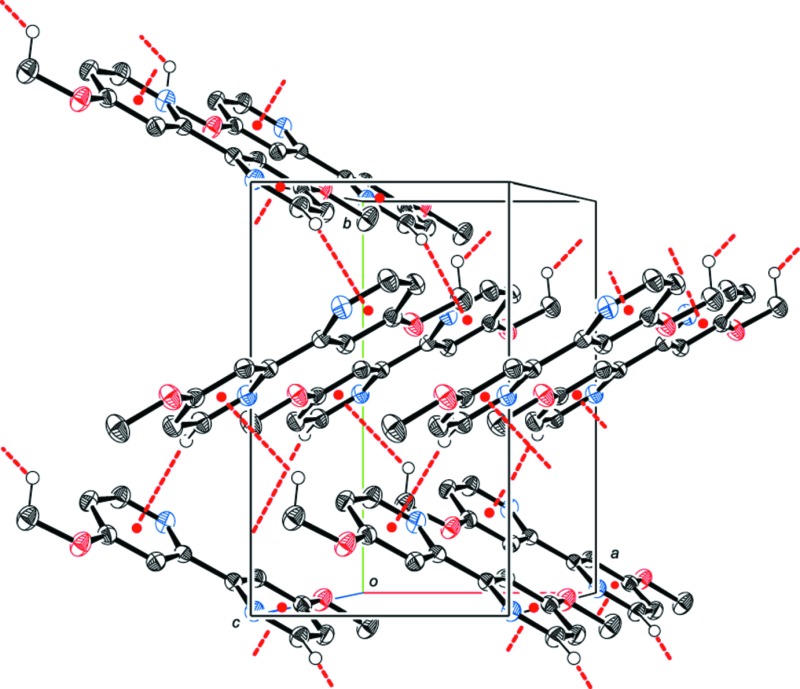
Part of the crystal structure showing the intersheet stacking interactions and the weak C-H···π hydrogen bonds.

. DOI: 10.1107/S2056989015013985/xu5860fig3.tif
Part of the crystal structure showing the inter­sheet stacking inter­actions and the weak C-H⋯π hydrogen bonds.

CCDC reference: 1414484

Additional supporting information:  crystallographic information; 3D view; checkCIF report

## Figures and Tables

**Table 1:** Hydrogen-bond geometry (, ) *Cg*1 and *Cg*2 are the centroids of the N1- and N2-rings, respectively.

*D*H*A*	*D*H	H*A*	*D* *A*	*D*H*A*
C11H11*B*N1^i^	0.98	2.58	3.503(3)	158
C12H12*B*N2^ii^	0.98	2.62	3.513(3)	151
C1H1*Cg*2^iii^	0.95	2.68	3.515(3)	146
C12H12*A* *Cg*1^iv^	0.98	2.72	3.439(3)	134

Symmetry codes: (i) 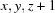; (ii) 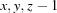; (iii) 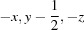; (iv) 
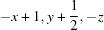
.

## supplementary crystallographic information

### S1. Comment

The molecular structure of title compound (I) is illustrated in Fig. 1. In the crystal, all bond lengths and angles are similar to those of the other similar 4,4'-substituted 2,2'-bi­pyridines (Merritt & Schroeder, 1956; Tynan *et al.*, 2003; Pearson *et al.*, 2004; Haberecht *et al.*, 2005; Fujihara *et al.*, 2005). The dihedral angle between two pyridine rings is 5.8 (1)°. The neighbouring molecules are linked *via* inter­molecular C–H···N inter­actions with an *R*_2_^2^(16) ring motif (Etter *et al.*, 1990; Bernstein *et al.*, 1995), generating 2D sheet structure as shown in Fig. 2. An overlapped arrangement of parallel pyridine rings in neighbouring molecules [centroid-centroid distance = 3.6655 (15) Å] is observed in the crystal structure. Furthermore, inter­molecular C–H···π inter­actions link the molecules into a three-dimensional network, as shown in Fig. 3.

### S2. Synthesis and crystallization

Crystals of (I) suitable for X-ray diffraction were obtained from solutions in acetone of a commercially available sample (Aldrich) by slow evaporation at 298 K. ^1^H NMR (500MHz, CDCl_3_): δ 4.10 (s, 6H, -OCH_3_), 7.03(dd, 2H, py-5H), 8.37(d, 2H, py-H3), 8.56(d, 2H, py-H6).

### S3. Refinement

The H atoms were included in calculated positions and treated as riding atoms: C—H = 0.95-0.98Å with *U*_iso_(H) = 1.5U_eq_(C) for methyl H atoms and = 1.2U_eq_(C) for aromatic H atoms.

### Crystal data

**Table:**

C_12_H_12_N_2_O_2_	*F*(000) = 228
*M**_r_* = 216.24	*D*_x_ = 1.369 Mg m^−^^3^
Monoclinic, *P*2_1_	Mo *K*α radiation, λ = 0.71073 Å
*a* = 6.4235 (11) Å	Cell parameters from 3527 reflections
*b* = 10.8139 (18) Å	θ = 2.7–28.3°
*c* = 8.0123 (14) Å	µ = 0.10 mm^−^^1^
β = 109.462 (2)°	*T* = 200 K
*V* = 524.76 (16) Å^3^	Plate, colourless
*Z* = 2	0.24 × 0.07 × 0.05 mm

### Data collection

**Table:**

Bruker APEXII CCD area-detector diffractometer	2301 independent reflections
Radiation source: Bruker TXS fine-focus rotating anode	2155 reflections with *I* > 2σ(*I*)
Bruker Helios multilayer confocal mirror monochromator	*R*_int_ = 0.014
Detector resolution: 8.333 pixels mm^-1^	θ_max_ = 27.1°, θ_min_ = 2.7°
φ and ω scans	*h* = −8→8
Absorption correction: multi-scan (*SADABS2014*; Bruker, 2014)	*k* = −13→13
*T*_min_ = ?, *T*_max_ = ?	*l* = −10→10
5925 measured reflections	

### Refinement

**Table:**

Refinement on *F*^2^	Hydrogen site location: inferred from neighbouring sites
Least-squares matrix: full	H-atom parameters constrained
*R*[*F*^2^ > 2σ(*F*^2^)] = 0.033	*w* = 1/[σ^2^(*F*_o_^2^) + (0.038*P*)^2^ + 0.1642*P*] where *P* = (*F*_o_^2^ + 2*F*_c_^2^)/3
*wR*(*F*^2^) = 0.084	(Δ/σ)_max_ < 0.001
*S* = 1.01	Δρ_max_ = 0.20 e Å^−^^3^
2301 reflections	Δρ_min_ = −0.16 e Å^−^^3^
147 parameters	Absolute structure: Flack *x* determined using 976 quotients [(I+)-(I-)]/[(I+)+(I-)] (Parsons *et al.*, 2013)
1 restraint	Absolute structure parameter: 0.7 (3)

### Special details

**Table:**

Geometry. All esds (except the esd in the dihedral angle between two l.s. planes) are estimated using the full covariance matrix. The cell esds are taken into account individually in the estimation of esds in distances, angles and torsion angles; correlations between esds in cell parameters are only used when they are defined by crystal symmetry. An approximate (isotropic) treatment of cell esds is used for estimating esds involving l.s. planes. Least-squares planes (x,y,z in crystal coordinates) and deviations from them (* indicates atom used to define plane) - 3.4311 (0.0049) x + 8.4883 (0.0065) y - 0.9456 (0.0066) z = 4.1170 (0.0067) * -0.0041 (0.0014) N2 * 0.0008 (0.0014) C6 * 0.0044 (0.0014) C7 * -0.0064 (0.0014) C8 * 0.0031 (0.0015) C9 * 0.0021 (0.0016) C10 -0.0086 (0.0030) O2 -0.0684 (0.0042) C12 Rms deviation of fitted atoms = 0.0039 - 3.4675 (0.0048) x + 8.7970 (0.0059) y - 0.1939 (0.0067) z = 4.4232 (0.0036) Angle to previous plane (with approximate esd) = 5.832 ( 0.127 ) * 0.0007 (0.0014) N1 * 0.0006 (0.0016) C1 * -0.0022 (0.0015) C2 * 0.0024 (0.0015) C3 * -0.0011 (0.0013) C4 * -0.0005 (0.0013) C5 0.0002 (0.0030) O1 -0.0031 (0.0045) C11 Rms deviation of fitted atoms = 0.0015 - 3.4311 (0.0049) x + 8.4883 (0.0065) y - 0.9456 (0.0066) z = 4.1170 (0.0067) Angle to previous plane (with approximate esd) = 5.832 ( 0.127 ) * -0.0041 (0.0014) N2 * 0.0008 (0.0014) C6 * 0.0044 (0.0014) C7 * -0.0064 (0.0014) C8 * 0.0031 (0.0015) C9 * 0.0021 (0.0016) C10 -3.3185 (0.0031) N1_$4 -3.3333 (0.0027) C1_$4 -3.4592 (0.0024) C2_$4 -3.5727 (0.0026) C3_$4 -3.5648 (0.0032) C4_$4 -3.4354 (0.0034) C5_$4 Rms deviation of fitted atoms = 0.0039 - 3.4675 (0.0048) x + 8.7970 (0.0059) y - 0.1939 (0.0067) z = 4.4232 (0.0036) Angle to previous plane (with approximate esd) = 5.832 ( 0.127 ) * 0.0007 (0.0014) N1 * 0.0006 (0.0016) C1 * -0.0022 (0.0015) C2 * 0.0024 (0.0015) C3 * -0.0011 (0.0013) C4 * -0.0005 (0.0013) C5 3.5634 (0.0029) N2_$3 3.4535 (0.0033) C6_$3 3.3280 (0.0034) C7_$3 3.3037 (0.0027) C8_$3 3.4295 (0.0025) C9_$3 3.5529 (0.0027) C10_$3 Rms deviation of fitted atoms = 0.0015

### Fractional atomic coordinates and isotropic or equivalent isotropic displacement parameters (Å^2^)

**Table:**

	*x*	*y*	*z*	*U*_iso_*/*U*_eq_	
C1	−0.1486 (3)	0.4462 (2)	0.0875 (3)	0.0267 (5)	
H1	−0.2534	0.4031	−0.0068	0.032*	
C2	−0.1727 (4)	0.4400 (2)	0.2528 (3)	0.0266 (5)	
H2	−0.2892	0.3940	0.2711	0.032*	
C3	−0.0204 (3)	0.5037 (2)	0.3910 (3)	0.0229 (4)	
C4	0.1487 (3)	0.56915 (19)	0.3571 (3)	0.0236 (4)	
H4	0.2556	0.6130	0.4492	0.028*	
C5	0.1577 (3)	0.56905 (19)	0.1864 (3)	0.0221 (4)	
C6	0.3359 (3)	0.63676 (19)	0.1424 (3)	0.0217 (4)	
C7	0.3547 (4)	0.6263 (2)	−0.0241 (3)	0.0238 (4)	
H7	0.2539	0.5768	−0.1126	0.029*	
C8	0.5240 (4)	0.68943 (19)	−0.0599 (3)	0.0235 (4)	
C9	0.6663 (4)	0.7627 (2)	0.0716 (3)	0.0268 (5)	
H9	0.7823	0.8081	0.0514	0.032*	
C10	0.6319 (4)	0.7668 (2)	0.2337 (3)	0.0295 (5)	
H10	0.7293	0.8166	0.3239	0.035*	
C11	−0.1939 (4)	0.4392 (3)	0.5974 (3)	0.0356 (6)	
H11A	−0.3380	0.4719	0.5251	0.053*	
H11B	−0.1765	0.4482	0.7230	0.053*	
H11C	−0.1846	0.3515	0.5698	0.053*	
C12	0.7110 (4)	0.7348 (2)	−0.2653 (3)	0.0321 (5)	
H12A	0.7009	0.8244	−0.2509	0.048*	
H12B	0.7005	0.7164	−0.3876	0.048*	
H12C	0.8526	0.7048	−0.1844	0.048*	
N1	0.0100 (3)	0.50795 (19)	0.0503 (2)	0.0244 (4)	
N2	0.4729 (3)	0.70612 (19)	0.2732 (2)	0.0275 (4)	
O1	−0.0221 (3)	0.50643 (16)	0.5595 (2)	0.0307 (4)	
O2	0.5337 (3)	0.67463 (15)	−0.2257 (2)	0.0304 (4)	

### Atomic displacement parameters (Å^2^)

**Table:**

	*U*^11^	*U*^22^	*U*^33^	*U*^12^	*U*^13^	*U*^23^
C1	0.0250 (10)	0.0285 (11)	0.0256 (10)	−0.0048 (10)	0.0069 (8)	−0.0029 (9)
C2	0.0252 (10)	0.0274 (11)	0.0292 (11)	−0.0049 (10)	0.0118 (9)	−0.0023 (10)
C3	0.0256 (10)	0.0222 (10)	0.0227 (10)	0.0027 (9)	0.0105 (8)	0.0002 (9)
C4	0.0224 (10)	0.0225 (10)	0.0242 (11)	−0.0003 (8)	0.0055 (8)	−0.0007 (9)
C5	0.0210 (9)	0.0208 (10)	0.0248 (10)	0.0015 (8)	0.0082 (8)	0.0014 (8)
C6	0.0201 (10)	0.0196 (10)	0.0252 (10)	0.0019 (8)	0.0071 (8)	0.0017 (8)
C7	0.0229 (10)	0.0234 (10)	0.0250 (10)	−0.0012 (8)	0.0077 (8)	−0.0016 (8)
C8	0.0239 (10)	0.0239 (11)	0.0235 (10)	0.0023 (9)	0.0090 (8)	0.0024 (8)
C9	0.0244 (10)	0.0279 (11)	0.0296 (11)	−0.0040 (9)	0.0112 (9)	0.0018 (9)
C10	0.0267 (11)	0.0338 (13)	0.0267 (11)	−0.0074 (9)	0.0071 (9)	−0.0046 (9)
C11	0.0397 (13)	0.0443 (14)	0.0292 (12)	−0.0120 (12)	0.0200 (10)	−0.0030 (11)
C12	0.0345 (12)	0.0370 (13)	0.0287 (12)	−0.0063 (10)	0.0158 (10)	0.0006 (10)
N1	0.0241 (8)	0.0265 (9)	0.0229 (9)	−0.0015 (7)	0.0081 (7)	−0.0003 (7)
N2	0.0257 (9)	0.0321 (10)	0.0252 (9)	−0.0040 (8)	0.0091 (7)	−0.0014 (8)
O1	0.0333 (8)	0.0362 (9)	0.0254 (8)	−0.0085 (7)	0.0137 (6)	−0.0025 (7)
O2	0.0325 (9)	0.0366 (9)	0.0256 (8)	−0.0094 (7)	0.0144 (7)	−0.0042 (7)

### Geometric parameters (Å, º)

**Table:**

C1—N1	1.332 (3)	C8—O2	1.359 (3)
C1—C2	1.385 (3)	C8—C9	1.390 (3)
C1—H1	0.9500	C9—C10	1.389 (3)
C2—C3	1.391 (3)	C9—H9	0.9500
C2—H2	0.9500	C10—N2	1.338 (3)
C3—O1	1.354 (3)	C10—H10	0.9500
C3—C4	1.398 (3)	C11—O1	1.436 (3)
C4—C5	1.388 (3)	C11—H11A	0.9800
C4—H4	0.9500	C11—H11B	0.9800
C5—N1	1.355 (3)	C11—H11C	0.9800
C5—C6	1.496 (2)	C12—O2	1.436 (3)
C6—N2	1.349 (3)	C12—H12A	0.9800
C6—C7	1.384 (3)	C12—H12B	0.9800
C7—C8	1.393 (3)	C12—H12C	0.9800
C7—H7	0.9500		
			
N1—C1—C2	125.1 (2)	C9—C8—C7	119.02 (19)
N1—C1—H1	117.4	C10—C9—C8	117.2 (2)
C2—C1—H1	117.4	C10—C9—H9	121.4
C1—C2—C3	117.7 (2)	C8—C9—H9	121.4
C1—C2—H2	121.2	N2—C10—C9	125.3 (2)
C3—C2—H2	121.2	N2—C10—H10	117.4
O1—C3—C2	124.60 (19)	C9—C10—H10	117.4
O1—C3—C4	116.61 (18)	O1—C11—H11A	109.5
C2—C3—C4	118.79 (19)	O1—C11—H11B	109.5
C5—C4—C3	118.80 (18)	H11A—C11—H11B	109.5
C5—C4—H4	120.6	O1—C11—H11C	109.5
C3—C4—H4	120.6	H11A—C11—H11C	109.5
N1—C5—C4	123.02 (19)	H11B—C11—H11C	109.5
N1—C5—C6	115.79 (17)	O2—C12—H12A	109.5
C4—C5—C6	121.18 (17)	O2—C12—H12B	109.5
N2—C6—C7	123.39 (19)	H12A—C12—H12B	109.5
N2—C6—C5	116.19 (17)	O2—C12—H12C	109.5
C7—C6—C5	120.41 (17)	H12A—C12—H12C	109.5
C6—C7—C8	118.92 (19)	H12B—C12—H12C	109.5
C6—C7—H7	120.5	C1—N1—C5	116.58 (18)
C8—C7—H7	120.5	C10—N2—C6	116.14 (19)
O2—C8—C9	125.1 (2)	C3—O1—C11	117.47 (18)
O2—C8—C7	115.88 (18)	C8—O2—C12	117.40 (17)
			
N1—C1—C2—C3	0.3 (4)	C6—C7—C8—C9	−1.1 (3)
C1—C2—C3—O1	−180.0 (2)	O2—C8—C9—C10	−179.9 (2)
C1—C2—C3—C4	−0.5 (3)	C7—C8—C9—C10	1.0 (3)
O1—C3—C4—C5	179.94 (18)	C8—C9—C10—N2	−0.1 (4)
C2—C3—C4—C5	0.4 (3)	C2—C1—N1—C5	−0.1 (3)
C3—C4—C5—N1	−0.2 (3)	C4—C5—N1—C1	0.0 (3)
C3—C4—C5—C6	−179.56 (19)	C6—C5—N1—C1	179.41 (18)
N1—C5—C6—N2	174.7 (2)	C9—C10—N2—C6	−0.5 (4)
C4—C5—C6—N2	−5.8 (3)	C7—C6—N2—C10	0.4 (3)
N1—C5—C6—C7	−5.5 (3)	C5—C6—N2—C10	−179.92 (19)
C4—C5—C6—C7	173.9 (2)	C2—C3—O1—C11	−0.2 (3)
N2—C6—C7—C8	0.4 (3)	C4—C3—O1—C11	−179.7 (2)
C5—C6—C7—C8	−179.28 (17)	C9—C8—O2—C12	3.0 (3)
C6—C7—C8—O2	179.70 (18)	C7—C8—O2—C12	−177.8 (2)

### Hydrogen-bond geometry (Å, º)

Cg1 and Cg2 are the centroids of the N1- and N2-rings, respectively.

**Table:**

*D*—H···*A*	*D*—H	H···*A*	*D*···*A*	*D*—H···*A*
C4—H4···N2	0.95	2.50	2.813 (3)	99
C7—H7···N1	0.95	2.46	2.789 (3)	100
C11—H11*B*···N1^i^	0.98	2.58	3.503 (3)	158
C12—H12*B*···N2^ii^	0.98	2.62	3.513 (3)	151
C1—H1···*Cg*2^iii^	0.95	2.68	3.515 (3)	146
C12—H12*A*···*Cg*1^iv^	0.98	2.72	3.439 (3)	134

Symmetry codes: (i) *x*, *y*, *z*+1; (ii) *x*, *y*, *z*−1; (iii) −*x*, *y*−1/2, −*z*; (iv) −*x*+1, *y*+1/2, −*z*.
