# Supplementary figures and images for: Crystal structure of 4,4′-dimeth­oxy-2,2′-bi­pyridine
Source: Acta Crystallogr E Crystallogr Commun. 2015 Jul 31;71(Pt 8):o623–4. doi: 10.1107/S2056989015013985 (PMC4571430; doi:10.1107/S2056989015013985)

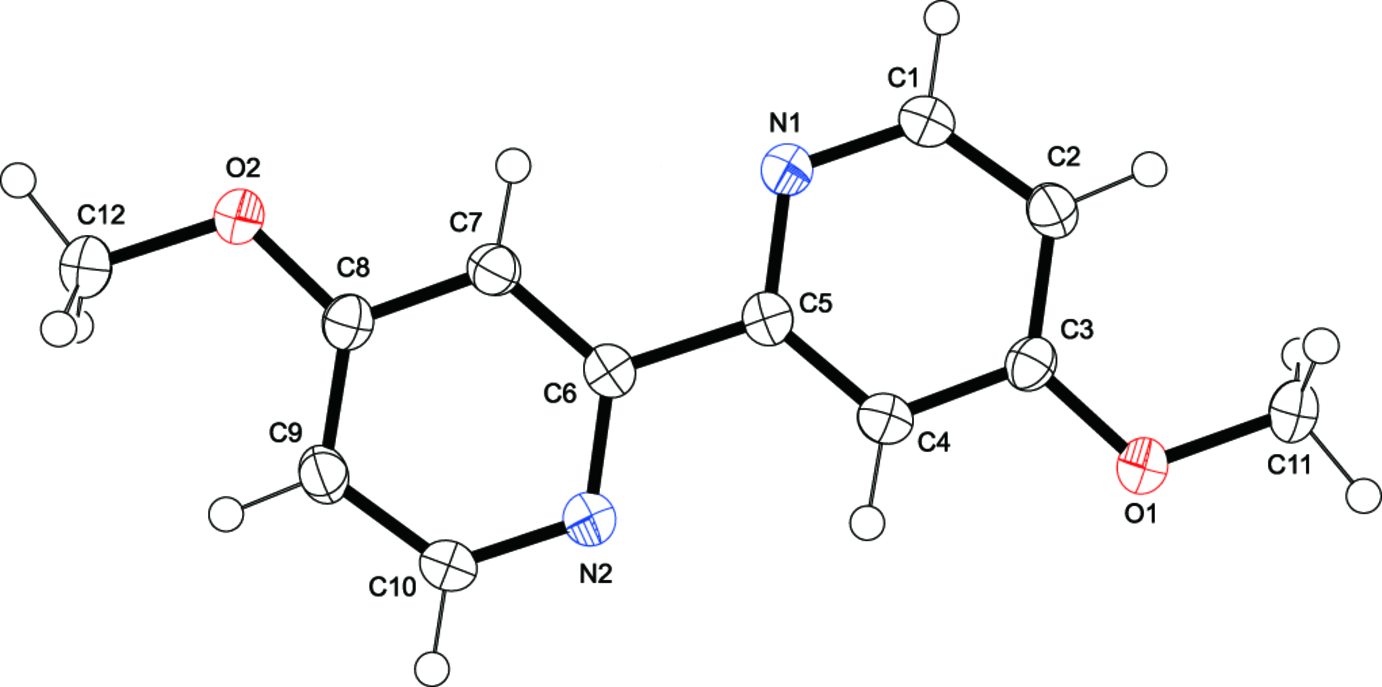

Supplement: Supplementary file 4 [file e-71-0o623-fig1.tif]

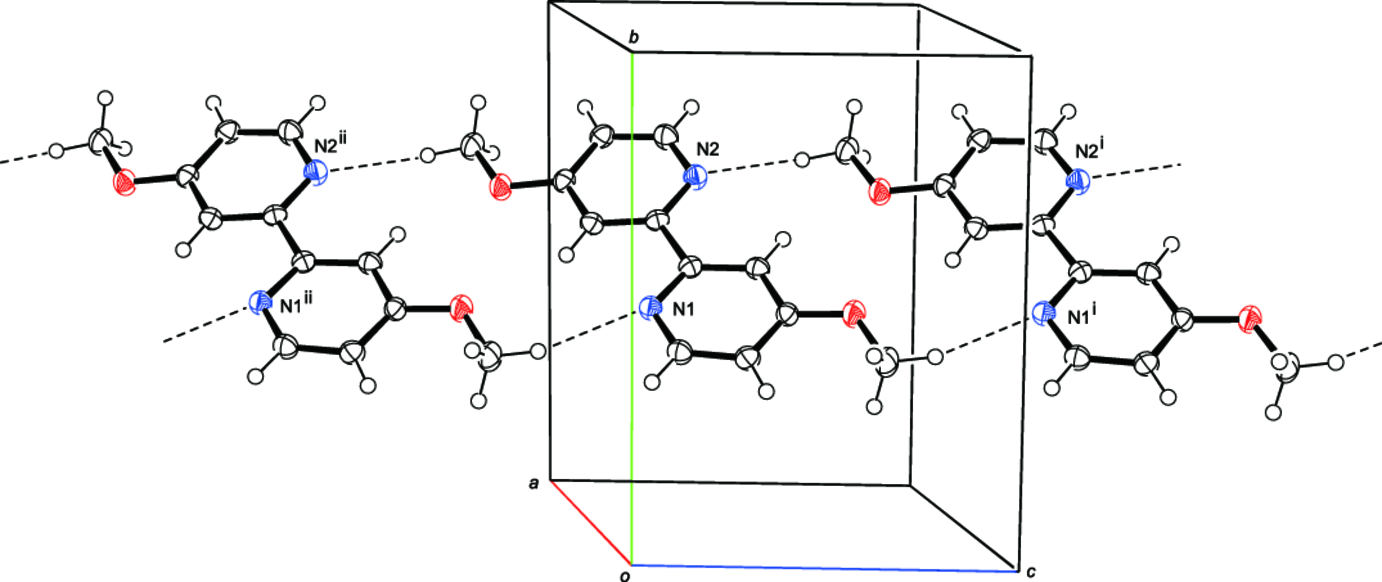

Supplement: Supplementary file 5 [file e-71-0o623-fig2.tif]

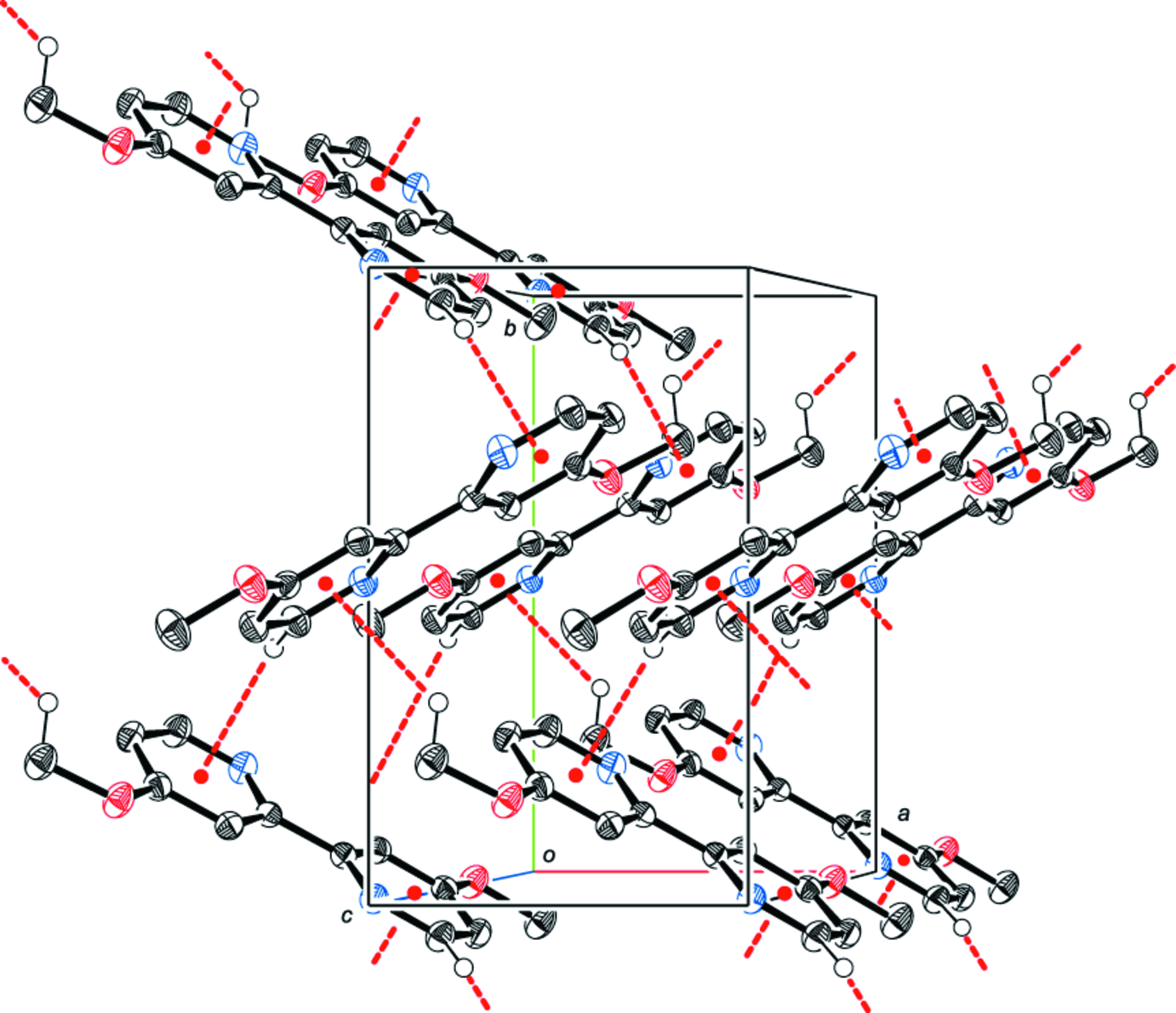

Supplement: Supplementary file 6 [file e-71-0o623-fig3.tif]
